# Ranking Cancer Risks of Organic Hazardous Air Pollutants in the United States

**DOI:** 10.1289/ehp.9884

**Published:** 2007-05-15

**Authors:** Miranda M. Loh, Jonathan I. Levy, John D. Spengler, E. Andres Houseman, Deborah H. Bennett

**Affiliations:** 1 KTL, National Public Health Institute, Department of Environmental Health, Kuopio, Finland; 2 Harvard School of Public Health, Department of Environmental Health, Cambridge, Massachusetts, USA; 3 University of Massachusetts, Lowell, Department of Work Environment, Lowell, Massachusetts, USA; 4 University of California, Davis, Department of Public Health Sciences, Davis, California, USA

**Keywords:** hazardous air pollutants, personal exposure, polycyclic aromatic hydrocarbons, risk assessment, volatile organic compounds

## Abstract

**Background:**

In this study we compared cancer risks from organic hazardous air pollutants (HAPs) based on total personal exposure summed across different microenvironments and exposure pathways.

**Methods:**

We developed distributions of personal exposure concentrations using field monitoring and modeling data for inhalation and, where relevant, ingestion pathways. We calculated risks for a nonoccupationally exposed and nonsmoking population using U.S. Environmental Protection Agency (EPA) and California Office of Environmental Health and Hazard Assessment (OEHHA) unit risks. We determined the contribution to risk from indoor versus outdoor sources using indoor/outdoor ratios for gaseous compounds and the infiltration factor for particle-bound compounds.

**Results:**

With OEHHA’s unit risks, the highest ranking compounds based on the population median are 1,3-butadiene, formaldehyde, benzene, and dioxin, with risks on the order of 10^−4^–10^−5^. The highest risk compounds with the U.S. EPA unit risks were dioxin, benzene, formaldehyde, and chloroform, with risks on a similar order of magnitude. Although indoor exposures are responsible for nearly 70% of risk using OEHHA’s unit risks, when infiltration is accounted for, inhalation of outdoor sources contributed 50% to total risk, on average. Additionally, 15% of risk resulted from exposures through food, mainly due to dioxin.

**Conclusions:**

Most of the polycyclic aromatic hydrocarbon, benzene, acetaldehyde, and 1,3-butadiene risk came from outdoor sources, whereas indoor sources were primarily responsible for chloroform, formaldehyde, and naphthalene risks. The infiltration of outdoor pollution into buildings, emissions from indoor sources, and uptake through food are all important to consider in reducing overall personal risk to HAPs.

The U.S. Clean Air Act designates hazardous air pollutants (HAPs) as those that “may reasonably be anticipated to be carcinogenic, mutagenic” (Clean Air Act Amendments 1990), and exhibit other adverse health effects. Effective reduction of exposures to HAPs requires determining the compounds, exposure pathways, and sources that contribute the most to human health risk.

Many prior risk assessments for HAPs have been limited by either including only indoor or outdoor concentrations or by examining only a small subset of carcinogenic HAPs. The U.S. Environmental Protection Agency (EPA) assessed the nationwide risk from outdoor concentrations of most of the HAPs. Based on the Assessment System for Population Exposure Nationwide (ASPEN) model (U.S. EPA 2000), the U.S. EPA found that almost half of total estimated lifetime cancer cases from HAPs could be attributed to volatile organic compounds (VOCs), with another 40% from polycyclic aromatic compounds (PAHs) (Woodruff et al. 2000). The median cancer risk was 17 cases of every 100,000 people. An updated assessment finds a median risk of 4 in 100,000 (U.S. EPA 2006a), accounting for changes in emissions estimates and lower cancer potency values for some of the larger contributors to risk (particularly 1,3-butadiene and formaldehyde). However, outdoor exposures account for only a portion of risk for many compounds.

In two older studies using indoor concentrations from homes and offices, one by Tancrede et al. (1987) and another by McCann et al. (1986), calculated cancer potency factors with data from animal and human studies. Tancrede et al. found annual mean risks from indoor air to be about 1 in 10,000 to 1 in 100,000, and McCann et al.’s risks are about an order of magnitude higher (McCann et al. 1986; Tancrede et al. 1987). Concentrations of many of these compounds, however, have changed since these studies were completed.

Personal exposure measurements from the Total Exposure Assessment Methodology (TEAM) studies provided estimates of individual cancer risks from benzene ranging from 1 in 10,000 for nonsmokers to 7 in 10,000 for smokers (Wallace 1991a). More recently, Payne-Sturges et al. (2004) found risks from personal exposure over three times higher than those calculated using the ASPEN modeled outdoor concentrations. Sax et al. (2006) also found risks from personal exposures of inner-city teenagers to be on the order of 1 in 10,000. Despite these studies, there has not been a broad analysis of cancer risk integrating total personal exposure to a wide range of organic HAPs in multiple microenvironments and across different exposure pathways. Also, two potentially high-risk classes of HAPs have not been included in previous personal exposure risk assessments—the dioxins and the PAHs.

Exposure to semivolatile HAPs, such as dioxins/furans and PAHs, can also come from noninhalation pathways, especially food ingestion (Butler et al. 1993; Ramesh et al. 2004; U.S. EPA 2003). Although these compounds are primarily released to the air, some fraction is bound to particulate matter and then deposited onto vegetation or water bodies where they build up in the food chain. Multimedia sampling has been done previously (Butler et al. 1993; Chuang et al. 1999), but only for a specific compound or class of compounds, and the risks of multi-pathway exposures have not been analyzed or compared across compound groups.

To gain a wider perspective on population risks from organic HAPs, we estimated the cancer risks in the United States by using calculated total personal exposure. We restricted ourselves to organic compounds that were responsible in aggregate for > 87% of the risk according to Woodruff et al. (2000), along with several others with known indoor sources or for which ingestion is a main route of exposure. We chose first to model baseline exposures, defined as those not including specifically known and consistent high exposure scenarios. We also examined situations for some compounds where a particular and relatively constant high exposure scenario can be developed.

We developed a flexible modeling framework that integrates data from different sources. The modeled personal exposure distributions were multiplied by a measure of cancer potency to calculate risk distributions that were ranked relative to each other. Because there is significant uncertainty in the toxicity estimates, we compared the risks calculated using two different sets of cancer potencies—the U.S. EPA’s Integrated Risk Information System (IRIS; U.S. EPA 2005) and the California Office of Environmental Health and Hazard Assessment (OEHHA; California Environmental Protection Agency 2005). In addition, we compared the proportion of risk attributable to indoor, outdoor, and ingestion exposures with the proportion of risk attributable to indoor and outdoor sources.

## Methods

In this analysis, we *a*) develop personal exposure distributions; *b*) calculate and compare baseline risks; *c*) examine the influence of alternative scenarios in exposure patterns and uncertainties in toxicity estimates on the results of the baseline assessment; *d*) determine the relative contribution of the ingestion pathway and the various inhalation microenvironments to the baseline risk; and *e*) disaggregate risk into indoor and outdoor source components.

Our baseline model represents a nonspecified population of office-working and nonemployed adults 18–65 years of age, which are assumed to be a relatively “low-exposure” population. We do not include smokers or manufacturing workers in the baseline because these populations are expected to have higher exposures from sources for which characterization was beyond the scope of this assessment. We classified compounds based on the availability of concentration data, emissions sources, and the primary route of exposure.

- Group 1 compounds are VOCs expected to come only from outdoor sources and include vinyl chloride, carbon tetrachloride, 1,3-dichloropropene, ethylene dibromide, and ethylene dichloride. Measured ambient concentrations are not readily available for most of these compounds.
- Group 2 compounds are VOCs with indoor and outdoor sources, and this group includes available data on concentrations in the home and other microenvironments. Group 2 includes benzene, formaldehyde, chloroform, 1,4-dichlorobenzene, methylene chloride, trichloroethylene, perchloroethylene, 1,3-butadiene, and acetaldehyde.
- Group 3 compounds are semivolatile, with a substantial amount of exposure from ingestion, and include PAHs and dioxins.

Because the results depend on the assumptions and choices for the input parameters, we conducted several analyses to examine the effect of variability in exposure and uncertainty in cancer potency values. We quantified exposure parameter variability associated with the baseline distribution, assuming that the higher percentiles of the distribution will encompass highly exposed individuals, except for cases where there is evidence of a bimodal distribution. In the latter case, there may be specific instances with additional indoor or outdoor sources, leading to a separate exposure distribution from the general population and, consequently, a different risk ranking. Regarding toxicity, cancer potency factors have not previously encompassed heterogeneity across the population; however, we examined uncertainty by comparing the results from two different sets of cancer potency factors.

### Exposure model

**Figure 1:**
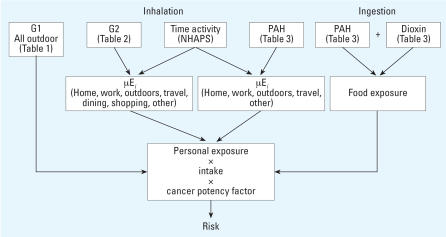
Representation of personal exposure and risk model. Refer to tables for compound concentrations. Abbreviations: G1, group 1 VOCs; G2, group 2 VOCs; NHAPS, National Human Activity Patterns Survey; μE_i_, exposure in microenvironment *i.*

Figure 1 illustrates the overall model used in this analysis. Exposure was calculated using Monte Carlo simulations in Crystal Ball (Decisioneering) according to Equation 1:


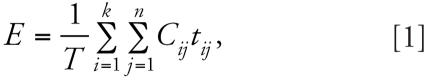


where *E* is the population exposure to pollutant X (summed over *k* individuals in *n* microenvironments); *C_ij_* is the concentration in the *j*th microenvironment for the *i*th individual; *t_ij_* is the time spent by the *i*th individual in the *j*th microenvironment; and *T* is the total amount of exposure time.

Distributions for the time spent in each microenvironment were taken from the National Human Activity Patterns Survey (NHAPS) (Graham and McCurdy 2004; Kleipeis et al. 2001; McCurdy and Graham 2003). The model population consisted of four types of people, sampled according to the percentage of the population that they represent in the 2000 Census (Clark and Weismantle 2003): nonworking males (11%), working males (38%), nonworking females (17%), and working females (33%). Because NHAPS provides only cross-sectional 24-hr data, each working person has the same weekday 5 days of the week and the same weekend day for 2 days. Nonworking individuals have the same day 7 days a week. The workday data came from people surveyed on a day they went to work, and weekend data came from working people surveyed on a day they did not go to work. To preserve the relationship between time in each microenvironment, we sampled from the NHAPS individuals’ diary-days directly. For the risk calculation we assume that these weeklong exposures are representative of lifetime exposures.

We derived concentration distributions by evaluating and combining data reported in several studies. We searched the peer-reviewed literature using the Science Citation Index (http://portal.isiknowledge.com/) for studies that measured the compounds of interest in each microenvironment, giving preference to studies conducted in the United States after 1995, to reflect more recent emission sources. Most of the data used were published before 2006. A small subset was obtained directly from the study investigators.

For group 1 compounds, we derived ambient concentration distributions from ASPEN model results. These concentrations were used as personal exposure concentrations because we assume no indoor sources for these compounds. ASPEN estimates ambient concentrations of HAPs for each census tract in the United States and includes emissions from point, area, and mobile sources, as well as secondary formation, decay, and deposition. ASPEN is the most comprehensive and spatially representative source of information for all outdoor HAPs concentrations. We used ambient concentrations for all census tracts based on 1996 emissions data, the most recent data available at the time this analysis was conducted (U.S. EPA 1996b) (Table 1).

For group 2 compounds, we compared each study’s reported parameters (usually the arithmetic mean ± SD, and median and 90th percentiles), the percentage of detectable samples, and the limits of detection, where reported. When deriving the final input distributions, we combined studies by weighting each city/geographic region equally. If multiple studies were conducted in an area, each study was given equal weight to determine the distribution in that city/geographic region. We assumed lognormal distributions for all studies where raw data were not available to us and fit the reported parameters in Crystal Ball (Table 2).

We derived in-home and outdoor concentrations from studies conducted in a range of urban and suburban communities, ranging from both coasts of the United States and the Midwest and Southwest, and including various ethnic groups and neighborhood sources (see Table 2). If > 50% of the values were under the detection limit, and the detection limit was deemed to be high compared with other studies, we chose not to use those data. In all other cases of low detects, we did not discard the study, because these values indicate a low environmental concentration level. We compared the studies qualitatively to assess whether a particular one appeared to indicate a higher or lower distribution than other studies, or if there appeared to be a sub-population with a distinctly different concentration distribution. Outdoor distributions were similarly developed.

Studies where the mean exceeded the 90th percentile (a highly skewed distribution) were not included in the baseline. Indoor data excluded were from Sax et al. (2004) from New York City in their summer sampling for 1,4-dichlorobenzene, and the National Human Exposure Assessment Survey (NHEXAS) trichloroethylene data from U.S. EPA’s Region 5 (Clayton et al. 1999). We also did not include any studies where the 90th percentile for 1,4-dichlorobenzene exceeded 100 μg/m^3^, which was > 100 times the median. Such a large tail indicated several homes from a few studies with extremely high concentrations, bringing the means to be almost 10 times the means of other studies (Adgate et al. 2004a; Sax et al. 2004).

Data for transportation, shopping, dining, and office workplaces were taken from various studies listed in Table 2. The miscellaneous “other” microenvironment was assigned the same concentration distributions as the outdoors.

Group 3 compounds consist of congeners that are weighted by a toxicity equivalence factor (TEF) relative to a reference congener: benzo[*a*]pyrene (BaP) for PAHs and for 2,3,7,8-tetrachlorodibenzo-*p*-dioxin (TCDD) for dioxins. We summed the TEF-weighted exposure concentrations for each congener to arrive at a total toxic equivalent (TEQ) concentration.

The PAHs were divided into two groups based on the evidence available for carcinogenic effects. PAHs in the first group (benzo[*a*]anthracene, benzo[*b*]fluoranthene, benzo[*a*]pyrene, chrysene, dibenzo[*a,h*]-anthracene, indeno[1,2,3-*cd*]pyrene) have evidence of carcinogenicity from animal studies, and we refer to them as PAH-B2. Concentrations in air and food were available for these compounds. Those in the second group (anthracene, benzo[*g,h,i*]perylene, phenanthrene, pyrene, fluoranthene) are less certain to be carcinogens and are named PAH-CD. We consider only the inhalation pathway for the PAH-CD compounds, because most of these compounds have not been as successfully quantified in food and have much smaller contributions to the total TEF-weighted food exposure than the PAH-B2 compounds. Naphthalene is treated separately because it has TEF-weighted concentrations that are at least an order of magnitude higher than other PAHs.

Data on home indoor and outdoor air PAH concentrations came from the Relationship of Indoor, Outdoor, and Personal Air (RIOPA) study in Los Angeles, California; Houston, Texas; and Elizabeth, New Jersey (Naumova et al. 2002). We combined reported gas- and particulate-phase PAH congener distributions to arrive at a total concentration distribution for each congener (Table 3). Naphthalene was not included in the above study; therefore, we used indoor data from other, smaller studies (Jia et al. 2005; Van Winkle and Scheff 2001). Several studies were excluded because there was a greater percentage of below-detection limit values, because of high detection limits or because the measurements were taken from studies much earlier than our time criteria. We included correlations in the indoors and outdoors for non-smoking homes to avoid artificially lowering the variability of the ultimate distribution, because many congeners have similar sources. Most studies did not report correlations between compounds, so we had to use a study conducted too early to be included in our overall distributions (Mitra and Ray 1995).

Because PAH concentrations decrease sharply within < 100 m from the road (Levy et al. 2003; Zhu et al. 2002), it is preferable, in the absence of commuter exposure data, to use roadside data to represent transportation microenvironments. Concentrations of specific congeners of PAHs in U.S. transportation microenvironments were not found in our literature search at the time of model development, so we used data from a roadside study in Denmark (Nielsen 1996). For congeners not reported in this study, we substituted the outdoor concentration (Table 3). Because little information on PAH concentrations exists for other microenvironments, except for naphthalene in offices, we used the outdoor concentration instead.

We derived PAH ingestion exposures from a study of BaP in food in the United States (Kazerouni et al. 2001) (Table 3). Because no other congeners were reported, we determined the contribution of BaP to total PAH in food by taking the ratio of each TEF-weighted PAH to BaP from several studies in Europe (Devos et al. 1990; Falco et al. 2003; Lodovici et al. 1995; Thomson and Muller 1998). We found that BaP is responsible for 30–80% of the TEF weighted mixture, with a mean value of 58%. We divided the BaP exposure in Kazerouni et al.’s (2001) U.S. study by this percentage.

Exposure to dioxins and dioxin-like compounds is dominated by ingestion, so inhalation exposures for dioxin were not included (Safe 1998; U.S. EPA 2003). We used the U.S. EPA evaluation of ingestion from surveys of dioxin concentrations in different foods and geographic areas and estimated intake rates from the *Exposure Factors Handbook* (U.S. EPA 1997) (Table 3).

### Industrial areas

Heavily industrial areas that are sources of group 1 compounds may not be adequately reflected in the tails of the general population exposure distributions. About 1% of all counties had median concentrations significantly higher than the medians of all other counties. A lognormal concentration distribution was fit based on the National Air Toxics Assessment (NATA; U.S. EPA 1996b) median and 95th percentile values for these counties (Table 1). We included compounds for which the ratio of the mean concentration of the top 1% counties to the baseline was > 2 (vinyl chloride and 1,3-dichloropropene) in the alternative scenario.

### 1,4-Dichlorobenzene

Homes with high levels of 1,4-dichlorobenzene in several studies were associated with users of moth repellants and/or deodorizers, representing a subset of the population with a separate 1,4-dichlorobenzene distribution and modeled as an alternative high exposure scenario (Table 2).

### Smoking

We derived the incremental exposure from environmental tobacco smoke (ETS) from Nazaroff and Singer (2004), who calculated a daily time-averaged concentration of for acetaldehyde, formaldehyde, benzene, and 1,3-butadiene attributed to ETS. For PAHs, we calculated the difference between smoking and nonsmoking home mean concentrations of PAHs (Mitra and Ray 1995).

### Risk calculation

We calculated risks by multiplying the intake of a substance by the cancer potency factor. The cancer potency factor has historically been a linear extrapolation from the high-dose animal or human studies to the low doses of environmental exposure using either a maximum likelihood estimate (for epidemiology) or the upper 95% confidence limit (for animal studies) on the dose response. We calculated baseline risk using OEHHA values, because these include compounds for which the U.S. EPA IRIS database does not have listed inhalation unit risks (Table 4). The TEQ exposures for group 3 compounds were multiplied by the cancer potency factor for the reference compound.

### Indoor and outdoor source contribution

To obtain the source contributions to exposure, we subtracted out the contribution to indoor concentrations from infiltration from the outdoors, and added this latter amount to the outdoor contribution. Exposure to group 1 VOCs was assumed to be the same indoors as outdoors, due to a lack of indoor sources and a penetration efficiency of 1 for gases (Lewis and Zweidinger 1992). Dioxins were assumed to have outdoor sources only. Microenvironments with indoor sources were home, work (office), shopping, and dining. Microenvironments classified with only outdoor source contributions were travel, the outdoors, the other nondefined micro-environments, and ingestion.

For gas-phase pollutants, we used the indoor:outdoor ratio to determine the indoor and outdoor contribution of each pollutant to the indoor concentrations. We calculated the fraction from indoor sources for the gas-phase and particle-phase PAHs separately. We used the phase distributions for each congener reported from the RIOPA study (Naumova et al. 2003) and assumed 100% infiltration efficiency for the gas-phase portion and an infiltration factor of 0.69 for the particle phase, from Meng et al. (2005) for the RIOPA data.

## Results

### Baseline risks

**Figure 2:**
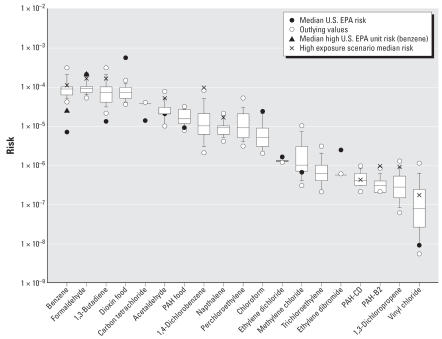
Baseline risk ranking using OEHHA toxicity estimates. 1,3-Dichloropropene does not have a unit risk value from OEHHA, so the U.S. EPA risk estimate was used. Symbols represent unit risks and measures and distribution medians. Smoking home exposure accounts for the high exposure for benzene, formaldehyde, 1,3-butadiene, acetaldehyde, naphthalene, and PAH-CD and PAH-B2. High home exposure from mothballs and associated products accounts for 1,4-dichlorobenzene. The top 1% emission counties are the high scenarios for 1,3-dichloropropene and vinyl chloride. Bars represent 5th and 95th percentiles. Boxes represent the 25th, 50th, and 75th percentiles

Figure 2 shows the baseline risk ranking using OEHHA unit risks or cancer potency factors, along with the median risk calculated using the U.S. EPA potency factors. Compounds with median risks falling near 1 × 10^–4^ are 1,3-butadiene, benzene, formaldehyde, all through inhalation, and dioxin through food. Compounds with risks between 1 × 10^–4^ and 1 × 10^–6^ include carbon tetra-chloride, acetaldehyde, PAHs through food, 1,4-dichlorobenzene, naphthalene, perchloroethylene, chloroform, and ethylene dichloride. For formaldehyde, dioxin, chloroform, and ethylene dibromide, calculation of risk using U.S. EPA’s potency factors resulted in higher values.

### Alternate exposure scenarios

Figure 2 also shows the median risk when using the alternative exposure scenarios for *a*) high ambient levels of 1,3-dichloropropene and vinyl chloride; *b*) exposure to ETS at home for 1,3-butadiene, formaldehyde, benzene, acetaldehyde, naphthalene, and other PAHs; and *c*) homes with extensive use of 1,4-dichlorobenzene products. For each of these cases, the additional risk was about an order of magnitude or less.

### Alternate toxicity

**Figure 3:**
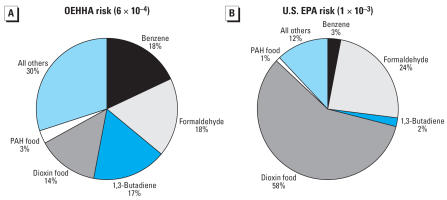
Risk from ingestion and inhalation. (*A*) Mean total risk calculated with OEHHA’s cancer potency values (6 × 10^−4^). (*B*) Median total risk using the federal U.S. EPA’s values (1 × 10^−3^). The inhalation fraction is broken down further into several of the higher-risk compounds.

A comparison of the mean risks by pathway and selected compounds using the OEHHA and U.S. EPA cancer potency factors is shown in Figure 3. Total risk using the OEHHA values is 6 × 10^−4^, compared with 1 × 10^−3^ using U.S. EPA values. Inhalation accounts for 83% and ingestion for 17% of total risk if the OEHHA values are used. Using U.S. EPA values, inhalation is assigned 41% of risk and 59% goes to ingestion, with dioxin responsible for 58% of total risk.

### Sources of exposure

If we compare the baseline mean risks from an exposure perspective, 69% of total risk comes from exposures occurring indoors (52% in the home), 9% from outdoors, 7% from travel time, and 15% from food. From a source rather than time–activity perspective, the distribution changes, where 35% of risk comes from indoor sources (27% in the home), 50% from outdoor sources, including mobile sources, and 15% from food. If we consider exposures from the PAHs and dioxin in food to come from either mobile or industrial sources, then the outdoor source contribution to risk becomes 65%.

**Figure 4:**
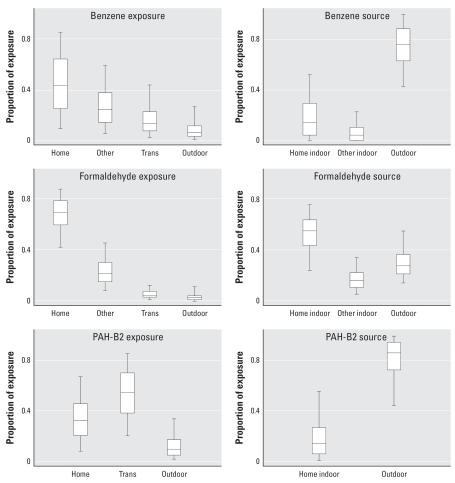
The contribution of exposure in microenvironments compared with indoor (home, offices, shops, and restaurants) and outdoor source contribution to inhalation risk for benzene, formaldehyde, and the TEF-weighted exposures to PAH-B2. Trans, transportation microenvironment. Bars represent 5th and 95th percentiles. Boxes represent the 25th, 50th, and 75th percentiles.

We also examined source contributions to inhalation exposure for the group 2 VOCs and the PAHs. Whereas group 2 VOCs and naphthalene had higher contributions from indoor exposures than outdoor exposures, the source contribution profiles differed depending on the compound (Table 5). Figure 4 shows more detail for benzene, formaldehyde, and PAH-B2. For benzene, exposures indoors at home and in other indoor microenvironments (work, shopping, dining) compose > 50% of exposure, on average. Benzene sources, however, are shown to be primarily (median of 80%) from the outdoors. Formaldehyde sources tend to be indoors, but outdoor sources are responsible for a median of 30% of formaldehyde exposure. For PAH-B2, transportation is responsible for the highest percentage of exposure, and the median contribution from outdoor sources is about 90%.

## Discussion

Average total lifetime cancer risk from organic hazardous air pollutants is about 6 in 10,000 when estimated using cancer potency factors determined by California’s OEHHA. The U.S. EPA’s factors lead to a risk estimate of about 1 in 1,000. Among the top-ranking compounds in both analyses are 1,3-butadiene, formaldehyde, benzene, and dioxin. Outdoor and indoor emissions as well as diet are all important contributors to total risk. By using cancer potency factors, our estimates likely represent upper-bound risks, but the internal consistency of our methodology allows us to compare among compounds and with other published studies.

### Comparison with other studies

We first placed our findings in context by comparing our results with those of previous risk assessments. When we examined median risks from our study using U.S. EPA cancer potencies and the 1996 NATA (U.S. EPA 1996b), for most compounds we calculated higher risks. We saw increases for compounds with indoor sources, such as formaldehyde and chloroform, as well as for some compounds with primarily outdoor sources, such as acetaldehyde, benzene, and 1,3-butadiene. Formaldehyde and acetaldehyde demonstrated the greatest differences in risk between personal and ASPEN exposure values. Some of the differences between the studies may be attributed to the tendency of ASPEN to underestimate concentrations when compared with ambient monitors (U.S. EPA 1996a). Compared with an earlier risk study by McCann et al. (1986), our median risks are lower, possibly because McCann’s data were from > 20 years ago, when some chemicals were used more widely, resulting in higher concentrations

A risk assessment using personal exposures measured for inner-city teenagers in Los Angeles and New York City provides a more direct comparison to our results for group 2 compounds. Sax et al. (2006) found that of the VOCs formaldehyde and 1,4-dichlorobenzene were the primary risk drivers. Their study had several high 1,4-dichlorobenzene homes, explaining the importance of this compound in this population. Our inclusion of high 1,4-dichlorobenzene homes shows a similar result, with this compound increasing in the risk ranking (Figure 2). Sax et al. (2006) also did an indoor/outdoor source apportionment using a mass balance model, producing similar percentage contributions to personal risks from indoor and outdoor sources from all matched compounds between our studies (Figure 4). These study similarities may partially be attributed to the fact that the study by Sax et al. (2006) was a primary source for the indoor/outdoor ratios we used, but our concentration inputs and time activity were for a much broader population than theirs. Our analysis found about the same mean percentage from indoor home sources and a higher percentage from outdoor sources, but we included additional indoor and outdoor microenvironments not distinguished by Sax et al. Our risks are also similar to those calculated by Payne-Sturges et al. (2004) using personal monitoring data in Baltimore.

### Uncertainties in cancer potency

Although we attempted to explore uncertainty in cancer potency factors by using OEHHA and U.S. EPA values, actual uncertainty in these values greatly exceeds the differences in the values used by these agencies. Some assumptions may systematically bias risk upward across all compounds. For example, the unit risk assumes a standard body weight (70 kg) and average breathing rate (20 m^3^/day), neither of which reflect the variability of the population at large (U.S. EPA 1997). Assumptions such as these may bias our estimates but would not change the ranking of compounds.

On the other hand, other assumptions could dramatically influence the risk estimates for individual compounds. In particular, for compounds for which the cancer-causing potential is attributed to cell death and proliferation rather than genotoxiciy, the linear at low dose assumption may not be applicable. Evidence for some compounds indicates a “threshold” rather than linear dose response, which implies that short-term high exposures could be important because of the cellular damage that could lead to cancer. Our analysis addresses only long-term chronic exposures, which is appropriate under the current linear framework for cancer potency estimation, but may need to be reevaluated in the future.

The U.S. EPA (2007) is currently reassessing formaldehyde based on studies that show that formaldehyde may follow a hockey-stick–or J-shaped dose response (Conolly et al. 2003, 2004). This is supported by the finding of a lack of increased formaldehyde in blood of the metabolized DNA protein cross-links in exposed rats (Heck and Casanova 2004). Also, some analyses have called into question the effect found in the occupational studies used to derive the formaldehyde risk (Heck and Casanova 2004; Marsh et al. 1992). Based on some of these arguments, the 1999 NATA uses a lower unit risk (3 orders of magnitude less then the IRIS value) for formaldehyde (U.S. EPA 2006b). Using this unit risk value, the formaldehyde risk, based on the median personal exposure in our model, drops to 9 × 10^−8^ from 2 × 10^−4^ (using the U.S. EPA risk) or 9 × 10^−5^ (using the OEHHA risk). Although the U.S. EPA considered formaldehyde a probable human carcinogen, in 2004, the International Agency for Research on Cancer (2004) deemed that there was sufficient evidence to consider formaldehyde a human carcinogen based on the epidemiology for nasopharyngeal cancer, in particular. This clearly demonstrates that there are potentially large uncertainties associated with interpretation of similar evidence, as well as ongoing changes in cancer potency estimation, making our risk rankings far from static.

Also being reassessed is chloroform, which has been found to be cytotoxic rather than genotoxic (Golden et al. 1997; Tan et al. 2003). Dioxin is also likely to be a tumor promoter rather than an initiator (Popp et al. 2006; Schwarz and Appel 2005). Questions regarding the main epidemiologic studies used in assessing dioxin risk relate to the method and difficulty of measuring and reconstructing exposure, the high levels of exposure, and the lack of quantification of potential exposure to other highly toxic compounds (Crump et al. 2003; Starr 2003).

Another interesting point with regard to toxicity is the difference in estimates for benzene provided by the U.S. EPA. The risks differ by almost a factor of 4 due to the difference in dose response predicted by two different exposure assessments of the same cohort. Benzene is the compound with the strongest human evidence for carcinogenicity, such that human epidemiology can be used to derive the cancer potency. However, we see that the estimated potency factor can depend on assumptions within the analysis, and is far from a defined quantity. Future work would benefit from the ability to better characterize the uncertainty surrounding model choices in the development of cancer potency factors.

### Uncertainties in exposure

Concentration data are lacking for nonhome microenvironments for many compounds, especially in workplaces and other indoor microenvironments, leading to greater uncertainty in these distributions. For example, although air risk from PAHs was not high in our risk ranking, we found that travel exposures may be important for this group. We were unable to find on-road or in-vehicle PAH congener data at the time of our analysis, so we used a Danish study. However, because diesel passenger cars are used more commonly in Europe, the U.S. PAH air mixture from mobile sources is probably different, particularly since diesel has been found to emit more of the lower-weight PAHs (Marr et al. 1999; Rogge et al. 1993; Shah et al. 2005; Westerholm and Hang 1994).

Group 1 compound concentrations were modeled, so we do not have ambient or in-microenvironment data for these compounds. We are assuming that ASPEN is providing a reasonable estimate of the potential exposures to group 1, and the high ends of these compounds’ distribution were still relatively low. Measurement data, however, would validate whether or not ASPEN is underpredicting concentrations for these compounds.

We were also limited by a small number of VOC studies in other microenvironments. Despite this, because the contribution to total exposure from the home drives risk for the baseline population, this data scarcity should not add a disproportionate amount of uncertainty for nonindustrial workers.

Uncertainties in PAH ingestion arise from the use of BaP intake values for the United States to extrapolate to total PAH food intake. We found that BaP exposure values in the United States, compared with those of several European countries, are about two to four times less. The variability of exposure through food can also be influenced by the distribution of foodstuffs, which can result in ingestion far from the source of environmental contamination; some types of cooking, particularly grilling meat (Kazerouni et al. 2001), which increases PAH concentration; and differences in intake rates.

The baseline exposure may not include specific groups of the population that may have a separate and much higher exposure distribution, such as people who are exposed to chemicals at work, live in a highly industrial region, or have large contributions from sources in their homes. In some cases, such as the group 1 compounds, even the counties with the highest 1% of modeled outdoor concentrations did not produce significant contributions to total risk. It is possible that we have underestimated exposure to group 1 compounds; however, the risk is so low from these compounds that the actual concentrations would have to be much higher for most of the group 1 compounds to confer high risks. In contrast, the subset of homes with a separate exposure distribution to 1,4-dichlorobenzene was highly exposed enough that it becomes a major risk driver for these households.

A key question, therefore, is what percentage of the population would fall into these high risk categories. According to an analysis of the National Health and Nutrition Examination Survey III (NHANES III) about 4% of a sub-sample of 982 subjects reported using mothballs, 9% reported toilet bowl deodorizer use, and 32% air freshener use. The first two products are the most likely sources of 1,4-dichlorobenzene, although some air freshener products may contain it. This study also found a higher probability of 1,4-dichlorobenzene product usage with nonwhites (Churchill et al. 2001), supported by findings from the TEAM studies in Los Angeles (Wallace 1991b) as well as studies finding higher 1,4-dichlorobenzene exposures among nonwhite participants (Adgate et al. 2004a; Sax et al. 2006). These percentages of the population may not be large, but the 1,4-dichlorobenzene risk becomes a significant risk driver. We note that there may also be higher naphthalene exposures among mothball users, but data were not sufficient to estimate this potential impact. Nonsmokers living with smokers—amounting to about 17% of households as of 1991 (U.S. Department of Health and Human Services 2006)—also have elevated risks, particularly from 1,3-butadiene. For the high exposure group 1 scenarios, the 1% of counties with the highest average concentrations includes high population counties, such that about 10–15% of the population live in these counties.

Other exposure assessment uncertainties pertain to the data for input distributions. Study methods can also influence the measurement of concentrations. Many studies (RIOPA, Minnesota, and NHEXAS) used passive charcoal badges, which have been shown to have high detection limits, and a negative bias in comparison to active sampling methods (Chung et al. 1999; Gordon et al. 1999). Although we did not notice large differences among studies with different methods (except for the percentage of nondetects), it is possible that there is some bias due to measurement methods.

Another uncertainty is that the concentrations in the model for group 2 compounds came from predominantly urban studies, with limited suburban data, and may not be representative of nonurban areas. Additionally, compounds from common sources, such as mobile sources, would exhibit high correlations, and therefore their concentrations would be expected to be related. We were able to incorporate correlations between PAH congeners, but we could not do so for other compounds.

One possibly high-risk HAP that was not included in our analysis is diesel exhaust. Diesel particulate matter is a significant exclusion from our HAPs list. A sample calculation using the 1999 NATA ambient concentrations for diesel PM and a recommended inhalation unit risk from OEHHA gives us a risk of 2.7 × 10^−4^, which is on the order of the dioxin risk. The difficulty with diesel PM is that it is more difficult to quantify in measurement studies, because usually elemental carbon is used but only as a proxy, so we chose to exclude it.

Additional uncertainties about the exposure assessment arise from the time–activity estimates and population assumptions. The time activity and exposures are calculated for 18- to 65-year-olds and extrapolated to a lifetime. We do not expect the differences for childhood and old age to be much greater than these adult exposures, although these omissions may create some bias in the results. We have tried to preserve the relationship between activities in broad categories across a day, but we were unable to create an accurate representation of long-term time–activity patterns. Our risks are based on the assumption that people’s week-long activities will not vary on average over time. On an individual level, this would misstate a person’s variability in time activity (i.e., by considering shopping/dining to occur either every day or on no days). When incorporated across the population, however, we would still be able to capture the population variability in activity patterns and therefore personal exposures. Although we do not expect that day-to-day behavior would exhibit large differences over time, future exposure modeling would benefit from the inclusion of longitudinal patterns of time use.

### Conclusions

In this analysis we attempted to estimate cancer risk from exposure to hazardous air pollutants to a general population, as well as high-risk scenarios for certain compounds. The risk to the general population is 2 orders of magnitude larger than the U.S. EPA acceptable risk level. Including risks from highly exposed and susceptible subpopulations would increase this risk. Because regulatory decisions are based on risk evaluations, it is important to know where exposures are coming from and to include as much of the current toxicologic information as possible. Our analyses provide insight not only about the high-risk compounds but also about the predominant sources of exposure for those compounds, which will allow for more effective means of exposure reduction. Future research should focus on refining toxicity evidence for the high-risk compounds in our analysis and on filling some identified microenvironmental exposure gaps, to further reduce uncertainties in decisions regarding prioritization among HAPs control measures.

## Correction

Equation 1 was incorrect in the original manuscript published online; it has been corrected here.

## Figures and Tables

**Table 1:** Exposure distributions for group 1 compounds (all lognormal).

Compound (μg/m^3^)^a^	GM	GSD
1,3-Dichloropropene	0.067	2.609
High 1,3-dichloropropene	0.23	1.13
Carbon tetrachloride	0.880	1.002
Ethylene dibromide	0.008	1.002
Ethylene dichloride	0.061	1.057
Vinyl chloride	0.001	4.966
High vinyl chloride	0.02	2.37

Abbreviations: GM, geometric mean; GSD, geometric standard deviation.

a All compound concentrations from U.S. EPA (1996b).

**Table 2:** Distributions of concentration inputs (μg/m^3^) for group 2 compounds [GM (GSD)].

Compound	Home (LogN)	Office (LogN)	Commute (LogN)	Dining distribution	Grocery (LogN)	Nongrocery (LogN)	Outdoor/other (LogN)
Formaldehyde	18 (2)^a^,^b^,^c^,^d^	15 (1.5)^e^	11 (1.5)^f^,^g^	Bet [1.4 (7.3)]^f^	14 (1.5)^f^	21 (2.7)^f^	2.5 (3.0)^a^,^b^,^d^
Acetaldehyde	9 (2)^a^,^c^	7.0 (1.6)^e^	4.3 (1.4)^f^,^g^	Gam [39 (0.9)]^f^	21 (2.1)^f^	10 (2.4)^f^	4.7 (1.4)^a^,^b^,^c^,^d^
1,3-Butadiene	0.3 (3.7)^a^,^d^	0.2 (3.4)^e^	1.5 (2.1)^f^,^g^	LogN [1.0 (6.3)]^f^	0.2 (3.4)^f^	0.2 (3.4)^f^	0.1 (3.6)^a^
Benzene	2.1 (3.1)^a^,^d^,^h^,^i^,^j^,^k^,^l^,^m^	3.5 (1.8)^e^	6.3 (1.9)^f^,^g^,^o^	LogN [3.1 (2.1)]^f^	1.7 (1.6)^f^	1.7 (2.1)^f^	1.8 (1.9)^a^,^d^,^i^,^j^,^k^,^m^
Methylene chloride	0.8 (5.8)^a^,^i^,^j^,^m^	0.7 (6.7)^e^	1.4 (2.0)^f^,^o^	LogN [1.4 (5)]^f^	1.1 (2.7)^f^	2.1 (5.8)^f^	0.4 (3.5)^a^,^d^,^i^,^j^,^m^
Chloroform	1.2 (2.8)^a^,^d^,^h^,^i^,^j^,^k^,^l^,^m^	0.3 (3.0)^e^	0.4 (2.4)^f^	Gam [1.9 (0.9)]^f^	1.2 (2.3)^f^	0.4 (3.7)^f^	0.2 (3.5)^a^,^d^,^h^,^i^,^j^,^l^,^m^
Trichloroethylene	0.2 (4.1)^a^,^h^,^i^,^j^,^m^	0.3 (4.0)^e^	0.3 (2.4)^f^,^o^	LogN [0.3 (5.2)]^f^	0.3 (2.1)^f^	0.4 (5.0)^f^	0.2 (2.5)^a^,^d^,^i^,^j^,^l^,^m^
Perchloroethylene	0.9 (4.3)^a^,^d^,^h^,^i^,^l^,^m^	2.0 (3.1)^e^	0.4 (2.5)^f^,^o^	LogN [2.1 (5.6)]^f^	0.9 (2.5)^f^	1.4 (3.4)^f^	0.4 (4.2)^a^,^d^,^i^,^j^,^k^,^l^,^m^
1,4-Dichlorobenzene	0.4 (6.9)^h^,^k^,^l^,^m^	0.9 (4.5)^e^	0.5 (2.6)^f^	LogN [1.5 (5.9)]^f^	2.7 (3.3)^f^	1.7 (7.7)^f^	0.1 (6.2)^a^,^d^,^l^,^m^
High 1,4-dichlorobenzene	18 (4.5)^a^,^i^						

Abbreviations: GM, geometric mean; GSD, geometric standard deviation. For offices and grocery stores, the nongrocery distribution was used for 1,3-butadiene. Distribution parameters: lognormal (LogN), GM (GSD), gamma (Gam), scale (shape), beta (Bet), alpha (beta).

a Sax et al. (2004).

b Reiss et al. (1995).

c Zhang et al. (1994).

d Weisel et al. (2005).

e BASE study data (Environmental Health and Engineering 2002; Girman et al. 1999).

f Loh et al. (2006).

g Rodes et al. (1998).

h Van Winkle and Scheff (2001).

i Adgate et al. (2004a).

j Payne-Sturges et al. (2004).

k Clayton et al. (1999).

l Adgate et al. (2004b).

m Sexton et al. (2004).

n Gordon et al. (1999).

o Batterman et al. (2002).

**Table 3:** Group 3 concentrations (μg/m^3^) and TEFs: lognormal distributions [GM (GSD)].

Compound	Home	Commute	Outdoor	TEF
PAH-B2				
Benzo[*a*]anthracene	3 × 10^−5^ (3.8)^a^	8 × 10^−5^ (2.5)^a^	8 × 10^−5^ (2.5)^a^	0.1^b^
Benzo[*b*]fluoranthene	1 × 10^−4^ (3.2)^a^	3 × 10^−4^ (2.1)^a^	3 × 10^−4^ (2.1)^a^	0.1^b^
Benzo[*a*]pyrene	6 × 10^−5^ (2.8)^a^	2 × 10^−3^ (2.0)^c^,^d^	9 × 10^−5^ (2.4)^a^	1^b^
Chrysene/Isochrysene	2 × 10^−4^ (2.5)^a^	3 × 10^−4^ (2.1)^a^	3 × 10^−4^ (2.1)^a^	0.001^b^
Dibenz[*a,h*]anthracene	8 × 10^−6^ (3.4)^a^	2 × 10^−5^ (1.9)^a^	2 × 10^−5^ (1.9)^a^	1^b^
Indeno[1,2,3-*c,d*]pyrene	1 × 10^−4^ (4.7)^a^	2 × 10^−3^ (2.1)^c^,^d^	3 × 10^−4^ (2.4)^a^	0.1^b^
PAH-CD				
Anthracene	1 × 10^−3^ (2.6)^a^	8 × 10^−4^ (2.0)^a^	8 × 10^−4^ (2.0)^a^	0.0005^e^
Benzo[*g,h,i*]perylene	2 × 10^−4^ (3.7)^a^	4 × 10^−3^ (1.8)^c^,^d^	3 × 10^−4^ (2.5)^a^	0.02^e^
Phenanthrene	3 × 10^−2^ (2.7)^a^	1 × 10^−3^ (1.8)^c^,^d^	2 × 10^−2^ (1.8)^a^	0.0005^e^
Pyrene	2 × 10^−3^ (2.8)^a^	2 × 10^−3^ (1.8)^a^	2 × 10^−3^ (2.1)^a^	0.001^e^
Fluoranthene	3 × 10^−3^ (2.3)^a^	2 × 10^−3^ (1.7)^a^	3 × 10^−3^ (2.2)^a^	0.05^e^
Naphthalene	9 × 10^−1^ (4.9)^f^,^g^	2 × 10^−1^ (3.0)^f^,^g^	1 × 10^−1^ (2.3)^f^,^g^	0.031^b^
Ingestion (mg/kg-day, TEF weighted)				
PAH	1.26 × 10^−6^ (1.54)^h^			
Dioxin	5.36 × 10^−10^ (1.55)^i^			

a Naumova et al. (2002).

b California EPA (2005).

c Nielsen (1996).

d Lim et al. (1999).

e Larsen and Larsen (1998).

f Van Winkle and Scheff (2001).

g Jia et al. (2005).

h Kazerouni et al. (2001).

i U.S. EPA (2003).

**Table 4:** Cancer unit risks and potency factors per μg/m^3^.

Compound	U.S. EPA	California (OEHHA)
1,3-Butadiene	3.00 × 10^−5^	1.70 × 10^−4^
Methylene chloride	4.70 × 10^−7^	1.00 × 10^−6^
Chloroform	2.30 × 10^−5^	5.30 × 10^−6^
Benzene (high/low for U.S. EPA)	7.8^a^/2.20 × 10^−6^	2.90 × 10^−5^
Carbon tetrachloride	1.50 × 10^−5^	4.20 × 10^−5^
Trichloroethylene	NA	2.00 × 10^−6^
Perchloroethylene	NA	5.90 × 10^−6^
1,4-Dichlorobenzene	NA	1.10 × 10^−5^
Formaldehyde	1.30 × 10^−5^	6.00 × 10^−6^
Acetaldehyde	2.20 × 10^−6^	2.70 × 10^−6^
1,3-dichloropropene	4.00 × 10^−6^	NA
Ethylene dibromide (central/high for U.S. EPA)	3.00/6.00 × 10^−4b^	7.10 × 10^−5^
Ethylene dichloride	2.60 × 10^−5^	2.10 × 10^−5^
Vinyl chloride (continuous adult)	4.40 × 10^−6^	NA
Vinyl chloride (continuous from birth)	8.80 × 10^−6c^	7.80 × 10^−5^
BaP (inhalation)	NA	1.10 × 10^−3^
BaP [oral slope factor (mg/kg/day) – 1]	7.3	12
Dioxin [oral slope factor (pg/kg/day) – 1]	1.00 × 10^−3^	1.30 × 10^−4^

NA, not applicable.

a Used higher estimate for comparisons.

b Used upper-bound estimate for comparisons.

c Used continuous from birth for comparisons.

**Table 5:** Median indoor source contributions to HAPs risk from inhalation exposure.

Indoor source	Percent
Acetaldehyde	15
Formaldehyde	70
1,3-Butadiene	10
Benzene	20
Chloroform	70
Methylene chloride	45
1,4-Dichlorobenzene	35
Perchloroethylene	30
Trichloroethylene	25
PAH-B2	10
PAH-CD	20
Naphthalene	60
